# Intestinal Angioedema: A Mimic of an Acute Abdomen

**DOI:** 10.7759/cureus.34619

**Published:** 2023-02-04

**Authors:** Nilkanth L Pal, Yasmin Fernandes

**Affiliations:** 1 Department of Radiology, Goa Medical College and Hospital, Bambolim, Goa, IND

**Keywords:** computed tomography, ultrasound, angioedema, visceral, intestinal, acute abdomen

## Abstract

Visceral angioedema presents with features mimicking an acute abdomen, causing a great challenge in the diagnosis of the disease thus delaying the treatment. A high degree of radiological suspicion and clinical correlation will help in identifying this less-known entity, avoiding unnecessary surgery. CT scanning is the preferred investigation, but concomitant ultrasonography improves the diagnostic efficacy of CT scanning.

## Introduction

Angioedema refers to edema of the cutis and mucosa of the upper airway or gastrointestinal tract secondary to extravasations of protein and fluid secondary to increased capillary permeability [1]. Bowel angioedema is a challenging diagnosis since it is a lesser-known entity and is not as frequently encountered as the angioedema of the face, tongue, genitals, extremities, or upper airways. Superficial angioedema presents with localized and transient edema which does not warrant radiological evaluation. However, angioedema of the gastrointestinal tract presents with abdominal pain mimicking an acute abdomen, which may lead to an unnecessary laparotomy. Hence, the radiological finding of localized or generalized bowel edema on sonography and/or CT scan and appropriate clinical evaluation help prevent unnecessary surgery. We present this case report to understand the imaging findings of this lesser-known entity to help with a timely and correct diagnosis and also to review other pathologies that may have similar findings.

## Case presentation

A 25-year-old patient with no prior co-morbidities presented to our emergency department with a history of severe abdominal pain, nausea, vomiting, and constipation for two days. Other relevant history provided by the patient included swelling over the face, generalized urticaria, and difficulty breathing. No significant past history or previous such episodes were documented. All vitals were stable (pulse: 84 beats per minute, blood pressure: 110/70 mmHg, and respiratory rate: 28 breaths per minute). Laboratory investigations were within normal limits. Physical examination revealed generalized abdominal guarding and tenderness. There was facial puffiness with edema of the lips and in the periorbital region. Wheals were seen on the skin over the abdomen, chest, and bilateral lower limbs. Indirect laryngoscopy showed a congested epiglottis, bilateral vocal cords, and arytenoids.

**Figure 1:**
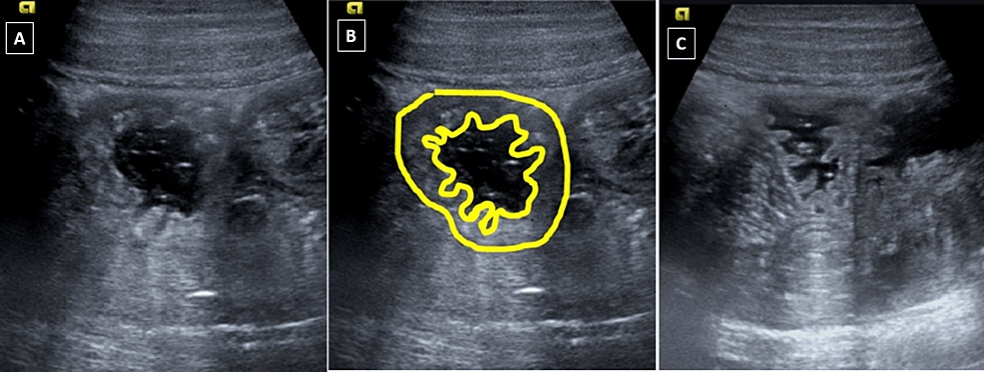
Bowel ultrasonography Figure A shows the bowel wall thickening with thickened mucosal folds. Figure B depicts the same image with the bowel outlined by markings. The inner line represents the mucosal surface, and the outer line represents the serosal surface. Figure C shows the complete resolution of the bowel edema on repeat ultrasonography performed 24 hours later.

Due to abdominal complaints, the patient was referred to the radiology department for ultrasonography. Ultrasound showed circumferential mural thickening of one of the proximal jejunal loops secondary to submucosal edema with thickened mucosal folds (Figure 1).

**Figure 2:**
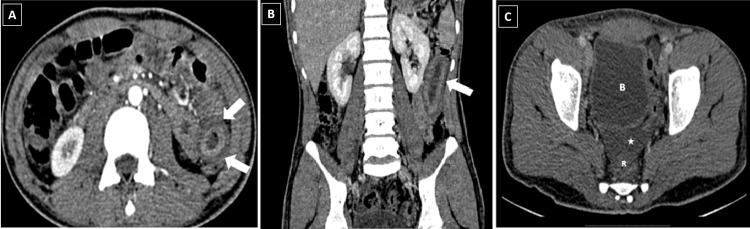
Contrast-enhanced CT scan Figure A (axial) and Figure B (coronal reformats) are contrast-enhanced CT scan images showing the presence of bowel wall thickening with submucosal edema producing the target appearance (arrows). Note the intense enhancement of the mucosal surface. This abnormality was segmental, involving one of the proximal jejunal loops. Figure C shows free fluid in the pelvis (star). B: bladder; R: rectum

This loop showed adequate peristalsis. Minimal free fluid was seen in the pelvis. A CT scan of the abdomen confirmed the findings of sonography by demonstrating submucosal edema in one of the proximal jejunal loops, causing circumferential mural thickening of the bowel and the target pattern of enhancement on the post-contrast scan (Figure 2).

There was stranding and fluid in the adjacent mesentery. There was no evidence of pneumatosis or pneumoperitoneum. Free fluid was also seen in the pelvis. In correlation with the patient’s clinical symptoms, small bowel thickening was suggested to be secondary to intestinal angioedema. The patient was started on steroids, and repeat ultrasonography 24 hours later showed a reduction in the mural thickening, confirming the diagnosis of intestinal angioedema. The patient was discharged three days later with a complete resolution of the symptoms.

## Discussion

Intestinal angioedema refers to edema in the submucosal space of the bowel that causes thickening of the bowel wall. Superficial angioedema presents with transient swelling and non-pitting edema; laryngeal edema may present with life-threatening respiratory distress [2], whereas gastrointestinal involvement presents non-specific symptoms such as abdominal pain, nausea, and vomiting that mimic an acute abdomen, leading to unnecessary laparotomies [3]. Rarely, bowel angioedema leads to hypovolemic shock due to the massive accumulation of fluid in the bowel lumen, its wall, and the peritoneum [4].

Angioedema is classified according to its cause, such as 1) allergic angioedema; 2) drug-induced, angiotensin-converting enzyme inhibitor (ACE-I)-mediated, and non-steroidal anti-inflammatory drug (NSAID)-mediated; 3) hereditary angioedema (HAE) with deficiency of the C1 esterase inhibitor enzyme; 4) acquired deficiency of the C1 esterase inhibitor enzyme; and 5) inherited angioedema with a normal C1 esterase inhibitor. Allergic angioedema is caused by a reaction to certain foods, insect bites, drugs, or environmental allergens. HAE is divided into two types: Type I occurs due to a deficiency of C1 esterase, while Type II occurs due to a dysfunctional enzyme [5]. Inherited angioedema results from mutations in the coagulation factor XII gene, leading to increased gene expression and subsequently elevated levels of bradykinin [6].

The mechanism of angioedema is not well understood, but most etiologies except allergic angioedema and non-steroidal anti-inflammatory drug (NSAID)-mediated angioedema involve bradykinin pathways and subsequent vasodilatation. The allergic type involves histamine pathways, and the NSAIDS reaction is mediated by the inhibition of cyclooxygenase (COX)-1, which results in the overproduction of vasoactive substances including cysteinyl leukotrienes [5].

Radiological investigations such as abdominal X-rays and fluoroscopy are not of particular help in diagnosis [7,8]. Ultrasonography (USG) shows the presence of bowel thickening secondary to submucosal edema, thickened mucosal folds (Figure 1), and free fluid. The involved bowel loop showed good peristalsis, thus helping to rule out ischemia (one of the differential diagnoses of intestinal angioedema). However, USG is operator-dependent, and findings may be obscured due to bowel gas. Hence, CT of the abdomen is the preferred investigation method.

CT findings of angioedema include bowel wall thickening secondary to submucosal edema, stranding and edema of the adjacent mesentery, and abdominal free fluid (Figure 2). These bowel loops appear distended secondary to the intraluminal accumulation of fluid. The submucosal edema produces the target pattern of enhancement on the post-contrast scan due to enhancement of the inner mucosa and outer muscularis or serosal layer with non-enhancement of the submucosa (Figure 2). The involved bowel shows regular mucosal fold thickening. These findings are usually segmental and transient. There is no associated lymphadenopathy or obstruction of the bowel [4,5,7].A repeat CT scan or ultrasonography will show the resolution of the bowel edema, confirming the diagnosis [9] (Figure 1C).

Our patient presented with facial and intestinal angioedema. We repeated the ultrasonography within 24 hours, which showed almost complete resolution of the mural thickening. We performed repeat ultrasonography instead of a second CT scan to avoid radiation. We could not establish the cause of angioedema in our patient. No family history or prior such episodes were documented by the patient. No known allergies or medication intake were noted. We were unable to perform C1 esterase inhibitor, C2, or C4 levels because these tests were not available at our institution (C2 and C4 are C1 esterase inhibitor substrates). These levels are abnormally low in hereditary angioedema (HAE) and angioedema secondary to acquired deficiency of the C1 esterase inhibitor enzyme.

Differential Diagnosis

These imaging findings of intestinal angioedema are similar to those seen with other causes of acute abdomen, i.e., bowel ischemia, intramural hemorrhage, vasculitis, inflammatory bowel disease like Crohn’s disease, lymphoproliferative disease, radiation enteritis, nephrotic syndrome with hypoproteinemia, infectious enteritis, and graft versus host disease [4,10]. For the diagnosis of intestinal angioedema, there needs to be a high degree of suspicion and clinical correlation. Ischemia due to arterial causes may show non-enhancement of the bowel lumen, vascular occlusion, or pneumatosis. It will be very difficult to differentiate angioedema from non-occlusive mesenteric ischemia, but the latter usually has low cardiac output, hypotension, or hypovolemia. Vasculitis may have cutaneous manifestations or known predisposing conditions such as systemic lupus erythematosus or Henoch-Schonlein purpura [10]. Intramural hemorrhage will have a history of anticoagulant intake with the occasional hyperdense appearance of the bowel wall on a plain CT scan [11]. There is an associated elevated prothrombin time and an international normalized ratio. Inflammatory bowel disease like Crohn’s produces multiple and skip areas of bowel wall thickening, luminal narrowing, and the associated "comb sign" due to the prominence of the vasa recti. Associated findings that will confirm Crohn’s disease are reactive adenopathy, creeping fat, abscesses, collections, and fistulae [12]. Lymphoma usually produces a homogenously thickened wall without a target or striated pattern of enhancement and has an irregular appearance. Lymphomas are frequently associated with multiple lymphadenopathies or involve other structures. Graft-versus-host disease is associated with organ transplantation and immunosuppression. A patient with radiation enteritis will have a history of irradiation and involvement of the bowel in the irradiated area. Patients with angioedema will have associated angioedema in other areas, as well as a family history or a history of previous such episodes. Symptoms and bowel edema are very transient, with a reduction of symptoms and edema sometimes within 24 hours. Hence, clinical history and detailed physical examination usually help in arriving at the correct diagnosis and eliminating unnecessary laparotomies.

## Conclusions

A high degree of suspicion will help in identifying this lesser-known entity. A detailed family and personal history, physical examination of the patient, and imaging features of bowel wall thickening with submucosal edema help in arriving at the correct diagnosis and preventing unnecessary surgical interventions. Combining abdominal ultrasonography findings with CT scan findings will increase the diagnostic value of CT for the diagnosis of intestinal angioedema.
